# Protein inhibitor of activated STAT3 reduces peripheral arthritis and gut inflammation and regulates the Th17/Treg cell imbalance via STAT3 signaling in a mouse model of spondyloarthritis

**DOI:** 10.1186/s12967-019-1774-x

**Published:** 2019-01-10

**Authors:** Hong-Ki Min, JeongWon Choi, Seon-Yeong Lee, Hyeon-Beom Seo, KyungAh Jung, Hyun Sik Na, Jun-Geol Ryu, Seung-Ki Kwok, Mi-La Cho, Sung-Hwan Park

**Affiliations:** 1 Division of Rheumatology, Department of Internal Medicine, College of Medicine, Seoul St. Mary’s Hospital, The Catholic University of Korea, Seoul, 137-070 South Korea; 2 Rheumatism Research Center, Catholic Research Institute of Medical Science, The Catholic University of Korea, Seoul, 137-040 South Korea; 3 Impact Biotech, Korea 505 Banpo-Dong, Seocho-Ku, Seoul, 137-040 South Korea; 4 Laboratory of Immune Network, Conversant Research Consortium in Immunologic Disease, College of Medicine, The Catholic University of Korea, Seoul, South Korea; 5 Rheumatism Research Center, Catholic Institutes of Medical Science, The Catholic University of Korea, 222 Banpo-Daero, Seocho-gu, Seoul, 137-701 South Korea; 6 Division of Rheumatology, Department of Internal Medicine, College of Medicine, Seoul St. Mary’s Hospital, The Catholic University of Korea, 222 Banpo-Daero, Seocho-gu, Seoul, 137-701 South Korea

**Keywords:** Spondyloarthritis, PIAS3, Type 17 helper T cell, Regulatory T cell

## Abstract

**Background:**

Spondyloarthritis (SpA) is chronic inflammatory arthritis, and interleukin (IL)-17 is crucial in SpA pathogenesis. Type 17 helper T (Th17) cells are one of major IL-17-secreting cells. Signal transducer and activator of transcription (STAT)-3 signaling induces Th17 differentiation. This study investigated the effects of protein inhibitor of activated STAT3 (PIAS3) on SpA pathogenesis. Curdlan was injected into SKG ZAP-70^W163C^ mice for SpA induction.

**Methods:**

The PIAS3 or Mock vector was inserted into mice for 10 weeks. Clinical and histologic scores of the paw, spine, and gut were evaluated. The expression of IL-17, tumor necrosis factor-α (TNF-α), STAT3, and bone morphogenic protein (BMP) was measured. Confocal microscopy and flow cytometry were used to assess Th cell differentiation.

**Results:**

PIAS3 significantly diminished the histologic scores of the paw and gut. PIAS3-treated mice displayed decreased expression of IL-17, TNF-α, and STAT3 in the paw, spine, and gut. BMP-2/4 expression was lower in the spines of PIAS3-treated mice. Th cell differentiation was polarized toward the upregulation of regulatory T cells (Tregs) and the downregulation of Th17 in PIAS3-treated mice.

**Conclusion:**

PIAS3 had beneficial effects in mice with SpA by reducing peripheral arthritis and gut inflammation. Pro-inflammatory cytokines and Th17/Treg differentiation were controlled by PIAS3. In addition, BMPs were decreased in the spines of PIAS3-treated mice. These findings suggest that PIAS3 could have therapeutic benefits in patients with SpA.

## Background

Sacroiliitis and spondylitis are the main symptoms of spondyloarthritis (SpA), which can be accompanied by inflammation in peripheral joints, enthesitis, gut inflammation, psoriasis, and uveitis. Various factors including environmental factors, genetic background, and immune dysregulation are involved in SpA pathogenesis. Interleukin (IL)-17 is a key pathologic cytokine in SpA pathogenesis, and various immune cells such as type 17 helper T (Th17) cells, natural killer T (NKT) cells, and γδ T cells secrete IL-17 [1].

IL-23 and IL-17 play pivotal roles in the development and progression of SpA [2, 3]. IL-23 is secreted from antigen-presenting cells, such as dendritic cells, and affects immune cells of the innate and adaptive immune systems to differentiate into IL-17-secreting immune cells [1, 2]. Although IL-17-secreting cells secrete not only IL-17, but also various pathologic cytokines, including IL-22, tumor necrosis factor alpha (TNF-α), and interferon (IFN)-γ, it is obvious that IL-17 is a key pathologic cytokine [2]. The IL-17-blocking monoclonal antibody, secukinumab, has already been approved for use in SpA patients [4, 5]. Th17 cells are a major source of IL-17, and an increased number of circulating Th17 cells was discovered in SpA patients [6, 7]. In contrast to IL-17-secreting cells, regulatory T cells (Tregs) maintain self-tolerance and play a suppressive role in autoimmune diseases [8]. Tregs in peripheral blood are decreased in ankylosing spondylitis (AS) patients relative to healthy controls [9, 10]. An imbalance in Th17 cells and Tregs is observed in SpA, and research studies have shown that potential therapeutic agents can ameliorate SpA symptoms by regulating the Th17/Treg cell imbalance [11, 12].

Naïve cluster of differentiation (CD) 4^+^ T cells can differentiate into various Th cells such as Th1, Th2, Th17, and Tregs. Signal transducer and activator of transcription (STAT) proteins act as essential transmitters of Th cell differentiation [13]. STAT3 is a regulatory factor that induces Th17 cell development from naïve CD4^+^ T cells [14]. Upon Th17 cell polarization, IL-23 plays a role in Th17 cell expansion and stabilization via the IL-23 receptor [15]. The expression of protein inhibitor of activated STAT3 (PIAS3) showed negative correlation with the level of Th17 in rheumatoid arthritis (RA) patients, and mice model of graft-versus-host disease revealed that PIAS3 upregulates Th17 population and downregulates Treg population [16, 17]. Blocking STAT3 signaling by PIAS3 may disturb SpA development by modifying Th17 cell and Treg cell differentiation.

The SKG ZAP-70^W163C^ mutation of the BALB/c mouse induces SpA features by curdlan injection [18]. A previous study showed that CD4^+^ cells extracted from curdlan-induced SpA mice transferred SpA features to mice with severe combined immunodeficiency (SCID) [18]. This finding supports the pathological role of the adaptive immune system in SpA pathogenesis. Therefore, curdlan-induced SpA mice are suitable models for investigating the mechanisms of therapeutic agents with regard to modulating the Th cell subtype.

This study determined the beneficial effects of a direct STAT3 inhibitor, protein inhibitor of activated STAT3 (PIAS3), in a mouse model of SpA. We measured the clinical and histologic scores, gut inflammation, and expression of cytokines in joints and gut tissues. We also determined the effects of PIAS3 on Th cell polarization by assessing Th17 and Treg cell populations to explain the mechanism of PIAS3 in SpA pathogenesis.

## Materials and methods

### Mice

SKG mice on a BALB/c background were kindly provided by Professor Shimon Sakaguchi (Department of Experimental Immunology, World Premier International Immunology Frontier Research Center, Osaka University). Mice were bred under specific-pathogen-free conditions and fed standard mouse chow (Ralston Purina, St. Louis, MO, USA) and water ad libitum. All of the experiments were assessed and approved by the Institutional Animal Care and Use Committee of the School of Medicine and the Animal Research Ethics Committee of the Catholic University of Korea, and were conducted in accordance with the Laboratory Animals Welfare Act, Guide for the Care and Use of Laboratory Animals.

### SpA induction and PIAS3 treatment

Curdlan (3 mg/kg) was injected intraperitoneally (i.p) into SKG mice aged 8–10 weeks. PIAS3 cDNA was obtained from Korea Human Gene Bank, Medical Genomics ResearchCenter, KRIBB, Korea. PIAS3 cDNA was subcloned to Kpn1 and Xho1 sties of pcDNA3.1 + (Invitrogen). The PIAS3 vector (100 µg) or Mock vector (100 µg) was inserted into SKG mice by electroporation and weekly hydrodynamic injection for 10 weeks according to previous research methods [16]. Clinical scores were measured weekly for the first 4 weeks and twice weekly for the following 6 weeks according to a previous study [18]. Scores of the affected joints were summed for each mouse.

### Histopathological analysis

Tissue samples (10% neutral-buffered formalin-fixed) from the peripheral joints, spine, colon, and small intestine were embedded in paraffin, and sections were cut at a thickness of 7 µm. The sections were dewaxed using xylene, dehydrated in an alcohol gradient, and stained with hematoxylin and eosin (H&E). H&E-stained sections of the peripheral joints and spines were scored for inflammation. The histologic scores of the peripheral joints and spine were calculated according to a previous study [18]. The histologic scores of the colon and small intestine were measured using a previously described method [19, 20].

### Immunohistochemistry

Immunohistochemical analyses were performed using the Vectastain ABC Kit (Vector Laboratories, Burlingame, CA, USA). Tissues were first incubated with primary antibodies (Abs) against IL-17, TNF-α, total STAT3, phosphorylated STAT3 (pSTAT3) s727, pSTAT3 y705, bone morphogenetic protein (BMP)-2, or BMP-4 (Santa Cruz Biotechnology, Santa Cruz, CA, USA) overnight at 4 °C followed by incubation with biotinylated secondary Abs against goat (Santa Cruz Biotechnology) and streptavidin–peroxidase complex (Vector Laboratories) for 1 h. The final colored product was developed using a chromogen (3,3-diaminobenzidine; Dako, Carpinteria, CA, USA). Two independent, blinded observers assessed all of the histologic scores. Images were taken using a DP71 digital camera (Olympus, Center Valley, PA, USA) attached to a BX41 microscope (Olympus) at a 3400× magnification. The positive cell was counted at high-power field (magnifications: 200×, 400×) with the aid of Adobe Photoshop software and averaged 3 randomly selected fields per tissue section.

### Confocal microscopy

Spleens were obtained from mice in both groups at 10 weeks after curdlan injection. To assess the differentiation of Th17 cells and Tregs, the spleen tissues were stained with Abs against CD4–fluorescein isothiocyanate (FITC), IL-17–phycoerythrin (PE), CD25–allophycocyanin, and forkhead box P3 (Foxp3)–PE (all from eBioscience, San Diego, CA, USA). Tissues were stained with Abs against CD4–FITC, *p*-STAT3–Y705–PE, *p*-STAT3–S727–PE, *p*-STAT5-PE, and PIAS3-PE (eBioscience) to examine the population of STAT-expressing cells. The stained tissue sections were visualized on a confocal microscope (LSM 510 Meta; Carl Zeiss, Oberkochen, Germany). Double or triple positive cells were counted in three high-power field (magnification: 200×) per section.

### Flow cytometric analyses

Cell pellets were prepared from the spleen tissues isolated from PIAS3- and Mock-treated mice. Populations of Th cells were examined by staining the tissues with a monoclonal Ab against CD4–peridin chlorophyll protein (perCP) and CD25-Allophycocyanin (APC) (eBioscience). Cells were permeabilized and fixed with CytoFix (BD Biosciences, San Jose, CA, USA) as instructed by the manufacturer, and stained with Abs against IL-17–FITC, IFNγ-PE, Foxp3-FITC (eBioscience), and PIAS3 APC (abcam, MA, USA). Flow cytometric analysis was primarily gated of CD4, then percentage of IL-17 positive, interferon-γ positive, Foxp3/CD25, and PIAS3 positive cells were calculated.

### Statistical analysis

All of the data are presented as the mean ± standard error of the mean. Statistical analyses were performed using SPSS 20.0 for Windows (IBM Corp., Armonk, NY, USA). Differences between two groups were analyzed using the Mann–Whitney test by assuming equal variance. *P* < 0.05 was considered statistically significant.

## Results

### Beneficial effects of PIAS on peripheral arthritis in mice with SpA

**Fig. 1:**
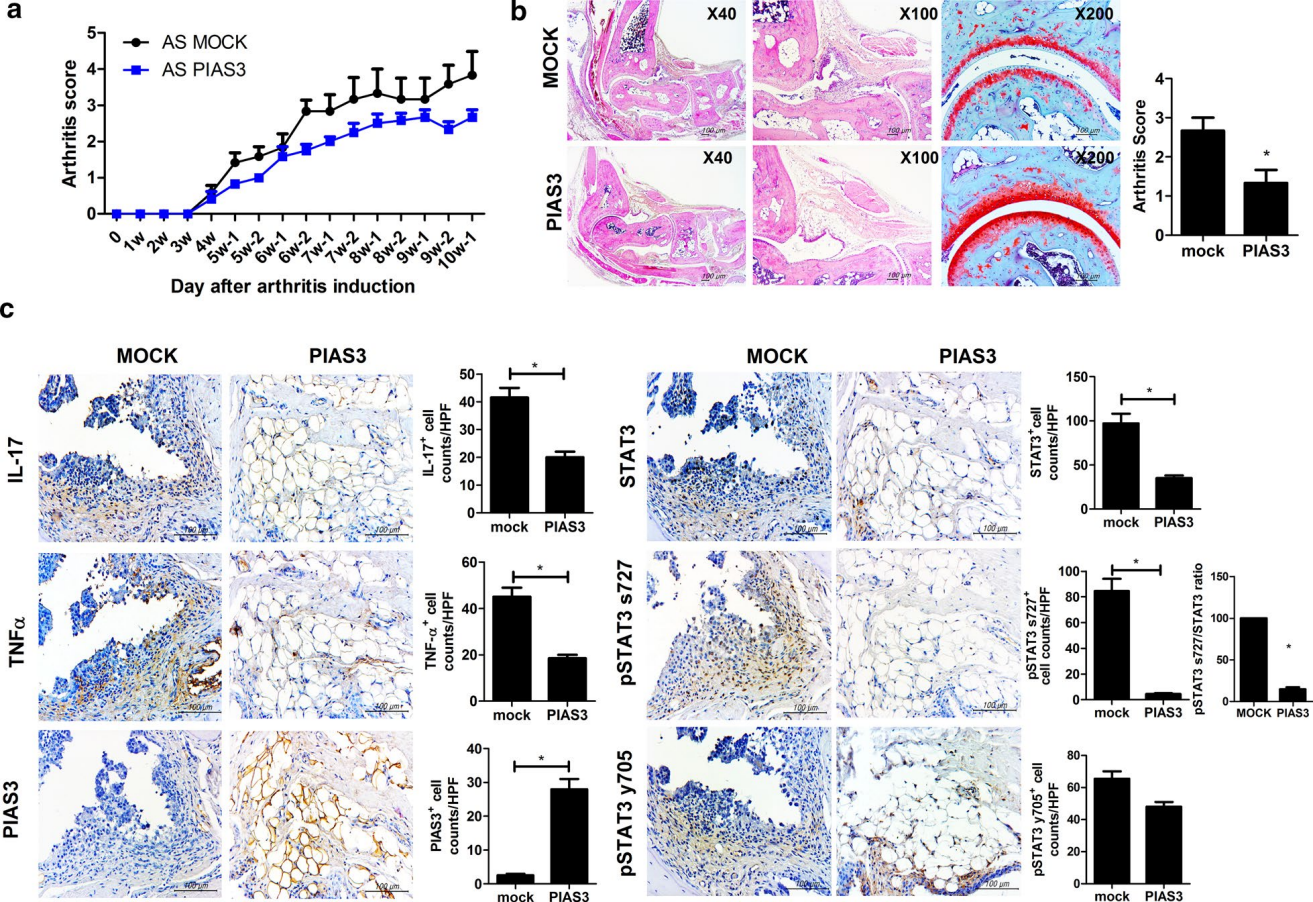
PIAS3 reduces peripheral arthritis, cartilage damage, and inflammatory cell infiltration in peripheral arthritis mice with spondyloarthritis (SpA). **a** The mean clinical score was determined based on arthritis severity. **b** Joint tissues were isolated from PIAS3 vector- or Mock vector-treated mice with SpA at 10 weeks after curdlan injection and stained with hematoxylin and eosin (H&E) and Safranin O. Arthritis score is shown in the bar graphs. **c** Peripheral joint tissues were stained with specific antibodies (Abs) against IL-17, TNF-α, STAT3, pSTAT3 s727, pSTAT y705, and PIAS3. Pro-inflammatory cytokine-, STAT3-, and PIAS3-positive cells are shown in the bar graphs. Data are represented as the mean ± standard error of the mean (SEM) of three independent experiments (*P < 0.05)

The clinical and histologic scores of peripheral arthritis were assessed to determine the preventive effects of PIAS3 on SpA mice. The clinical scores of PIAS3-treated mice showed tendency to be suppressed than Mock-treated mice (n = 5 for each group, Fig. 1a). The arthritis score (histologic score of peripheral joints) of PIAS3-treated mice was significantly lower than that of Mock-treated mice, and a gross histology image revealed that the joint structure of PIAS3-treated mice was more preserved than that of Mock-treated mice (Fig. 1b). The pro-inflammatory cytokines IL-17, and TNF-α were substantially suppressed in PIAS3-treated mice. Total STAT3, pSTAT3 s727, and ratio of pSTAT3 s727 to total STAT3 were effectively suppressed in PIAS3-treated mice (Fig. 1c). These results indicate that PIAS3 attenuates the induction of peripheral arthritis in mice with SpA by reducing STAT3 signaling and the secretion of pro-inflammatory cytokines.

### PIAS3 reduces pro-inflammatory cytokines, STAT3, and BMP expression in the axial joints of mice with SpA

**Fig. 2:**
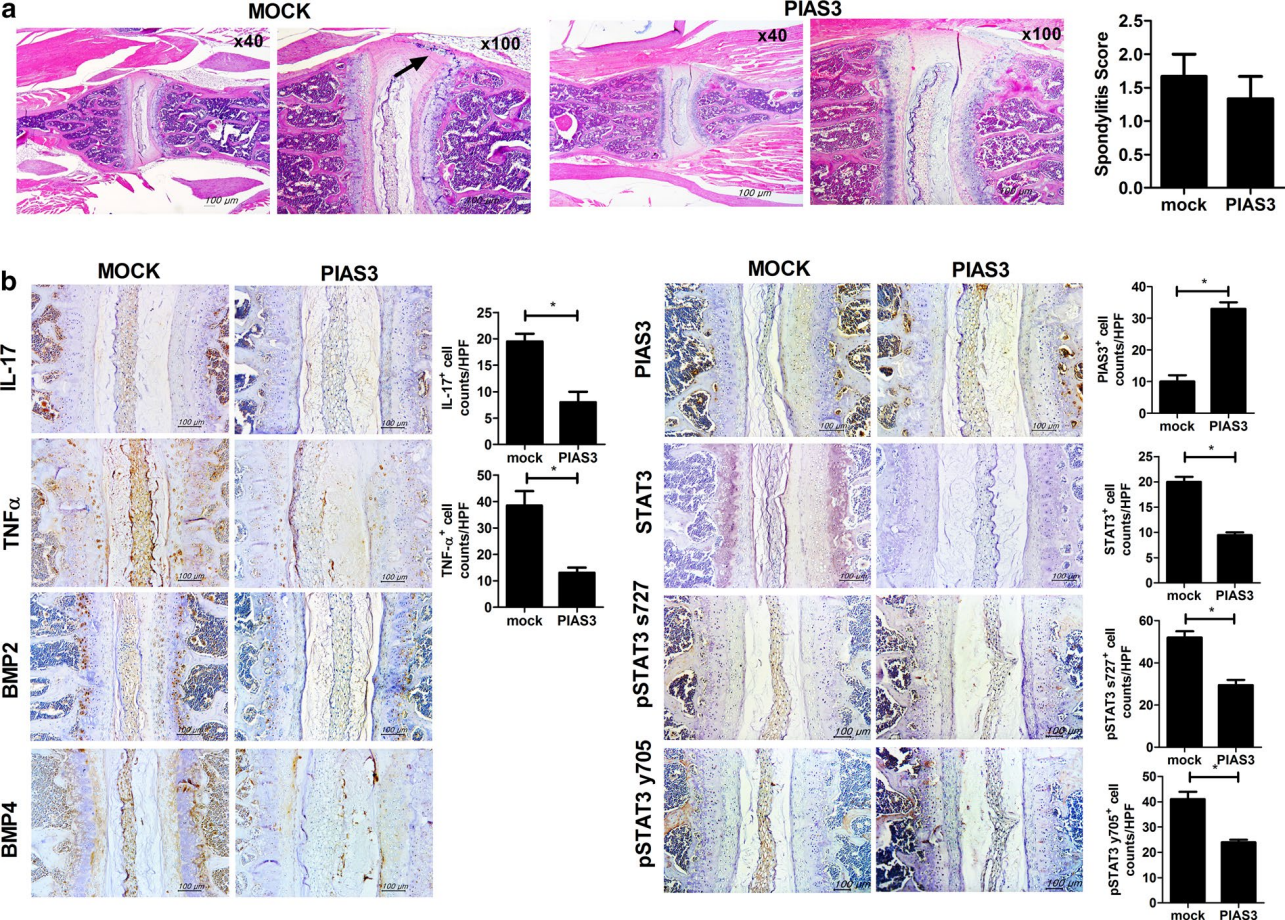
PIAS3 attenuates inflammatory cell infiltration in the spines of mice with SpA. **a** Spine tissues were isolated from PIAS3 vector- or Mock vector-treated mice with SpA at 10 weeks after curdlan injection and stained with H&E (black arrow = inflammatory cells). Spondylitis score is shown in the bar graphs. **b** Spine tissues were stained with specific Abs against IL-17, TNF-α, STAT3, pSTAT3 s727, pSTAT y705, BMP2/4, and PIAS3. PIAS3 suppresses the expression of pro-inflammatory cytokines, STAT3 and BMP2/4 in the affected spines of mice with SpA. Pro-inflammatory cytokine-, STAT3-, and PIAS3-positive cells are shown in the bar graphs. Data are represented as the mean ± SEM of three independent experiments (*P < 0.05)

Spine tissues were obtained from each group to assess the histologic scores and for immunohistochemical staining (n = 5 for each group). The spondylitis score (histologic score of the spine) of PIAS3-treated mice showed tendency to be suppressed than Mock-treated mice (Fig. 2a). PIAS3-treated mice displayed significant suppression of the pro-inflammatory cytokines, BMP-2/4, total STAT3, and pSTAT3 (Fig. 2b). Although spondylitis was not completely prevented by PIAS3 in mice with SpA, it could effectively reduce molecules involved in inflammation and osteoproliferation of the spine in mice with SpA.

### Beneficial effects of PIAS3 on gut inflammation in mice with SpA

**Fig. 3:**
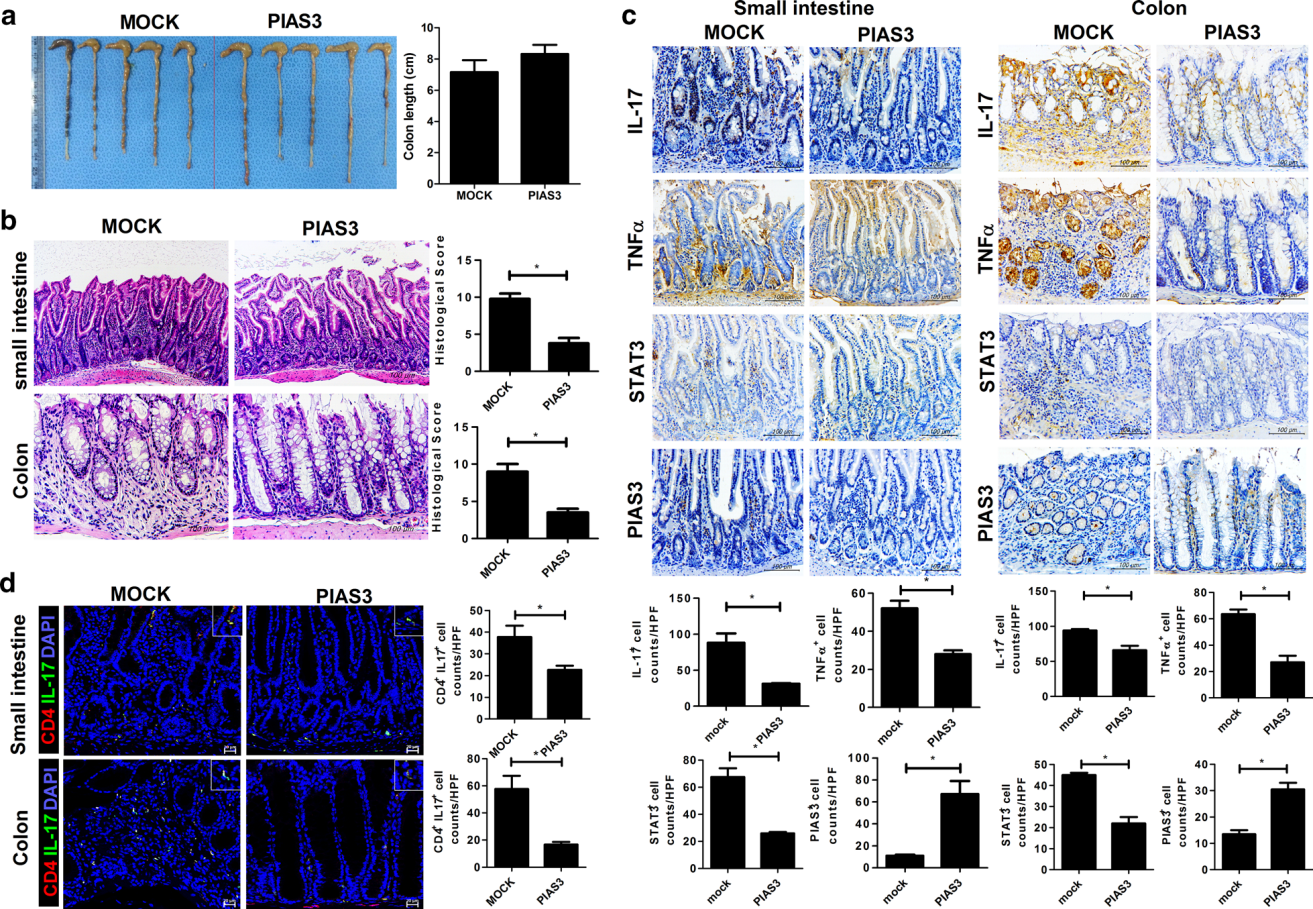
PIAS3 reduces gut inflammation in mice with SpA. **a** Gut length is shown in the bar graph. **b** Colon and small intestine tissues were stained with H&E. **c** Small intestine and colon tissues were stained with specific Abs against IL-17, TNF-α, STAT3, and PIAS3. Pro-inflammatory cytokine-, STAT3-, and PIAS3-positive cells are shown in the bar graph. **d** Colon and small intestine were stained with specific Abs against CD4 (red) and IL-17 (green). Double-positive cells are shown in the bar graph. Data are represented as the mean ± SEM of three independent experiments (*P < 0.05)

Gut inflammation was assessed by measuring the total gut length, colitis score, ileitis score, and cytokine expression in gut tissues. The difference in total gut length was minimal between the two groups; however, both ileitis and colitis scores were significantly lower in PIAS3-treated mice (Fig. 3a, b). Upon immunohistochemical staining of the gut, the expression of IL-17, TNF-α, and total STAT3 was significantly reduced in PIAS3-treated mice relative to Mock-treated mice (Fig. 3c). On confocal staining analysis, Th17 population was significantly suppressed in PIAS3-treated mice (Fig. 3d). These findings are important because the SpA model from SKG mice exhibits Crohn’s disease-like features, indicating that PIAS3 attenuates the progression of gut inflammation by regulating the STAT3 pathway.

### Effects of PIAS3 on Th17 and Treg cell differentiation in spleen

**Fig. 4:**
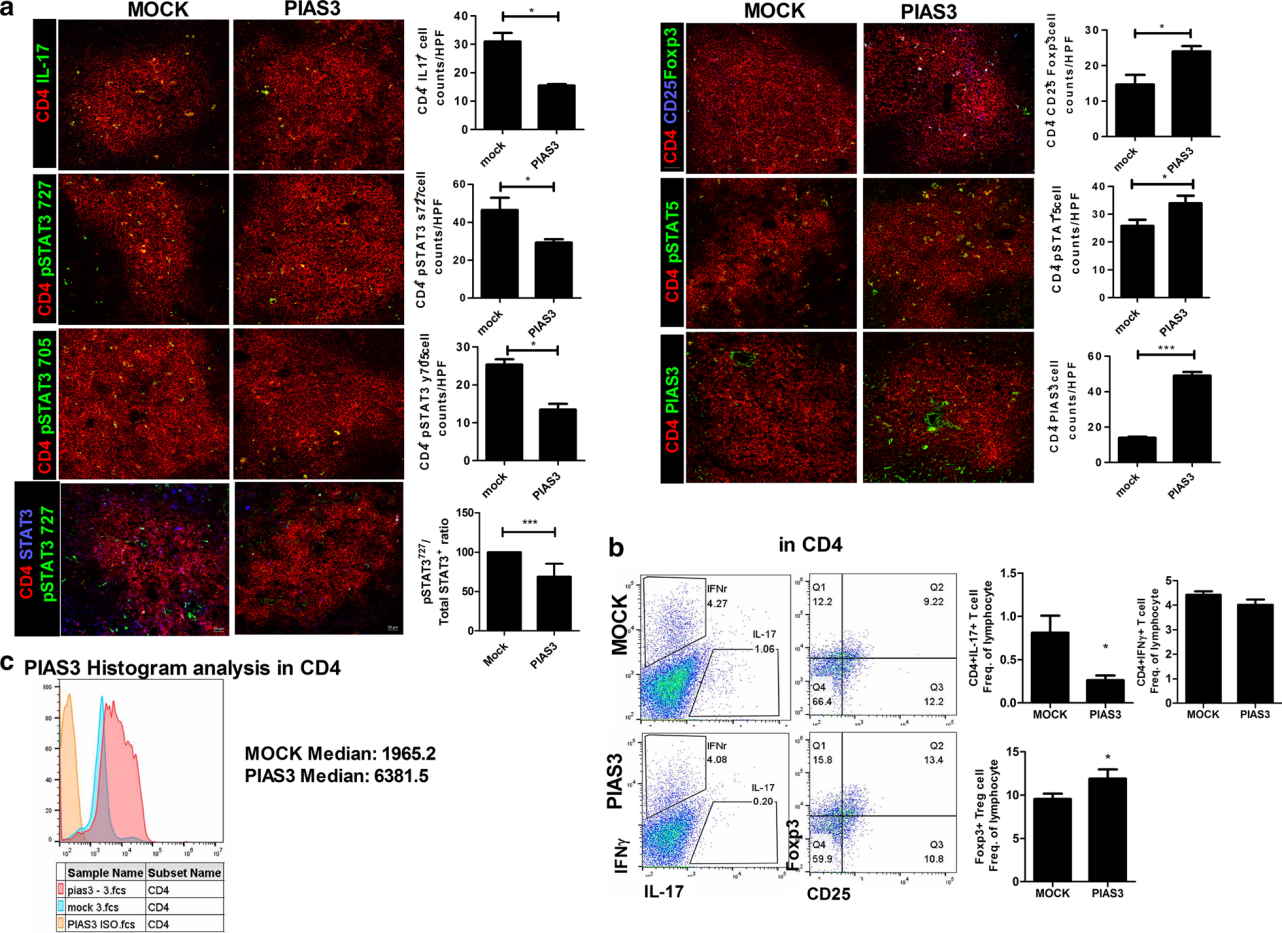
PIAS3 reciprocally regulates the Th17/Treg cell imbalance. Spleen tissues were isolated from mice in each group at 10 weeks after curdlan injection. **a** Tissues were stained with specific Abs against CD4 (red), CD25 (blue), IL-17 (green), FOXP3 (green), p-STAT3 y705 (green), p-STAT3 s727 (green), p-STAT5 (green), and PIAS3 (green). Positive cells are shown in the bar graphs. **b, c** Splenocytes were stained with Abs against CD4–PerCP, IL-17–FITC, IFNγ-PE, CD25-APC, FOXP3–FITC, and PIAS3-APC to determine the presence of Th17, Tregs and PIAS3 expression on CD4^+^ T cells. Data are expressed as the mean ± SEM of three independent experiments (*P < 0.05, ***P < 0.001)

After sacrifice, spleen tissues were obtained to assess Th cell differentiation. The number of Th cells was counted, and CD4^+^IL-17^+^, CD4^+^*p*-STAT3(S727)^+^, CD4^+^*p*-STAT3(Y705)^+^ cells, and ratio of CD4^+^*p*-STAT3(S727)^+^ cells to CD4^+^STAT3^+^ cells were significantly diminished in PIAS3-treated mice relative to Mock-treated mice, whereas CD4^+^CD25^+^FOXP3^+^ and CD4^+^*p*-STAT5^+^ cells were increased in PIAS3-treated mice relative to Mock-treated mice (Fig. 4a). Flow cytometric analysis showed downregulation of Th17 and upregulation of Treg in PIAS3-treated group than Mock-treated group (Fig. 4b, n = 11 for each group). Th1 differentiation did not show difference between two groups (Fig. 4b). PIAS3 was well encoded in CD4^+^ cell of spleen (Fig. 4c).

## Discussion

This study proved that PIAS3 has inhibitory effects on the progression of SpA in the aspect of peripheral arthritis and gut inflammation. The histologic scores of peripheral joints, the small intestine, and colon were all decreased in PIAS3-treated mice relative to Mock-treated mice. In addition, the expression of pro-inflammatory cytokines in peripheral joints and the gut was effectively reduced by PIAS3 treatment in mice with SpA. Treatment with PIAS3 decreased the expression of pro-inflammatory cytokines and osteoproliferative molecules in the axial joints. These findings could be explained by several mechanisms. First, PIAS3 decreased IL-17 and TNF-α secretion in the joints and gut. Second, PIAS3 regulated Th17 and Treg cells in the spleen.

IL-23 and IL-17 play crucial roles in the pathogenesis of SpA [20]. The major sources of IL-23 are monocytes and dendritic cells, and IL-23 controls immune cells of the adaptive and innate immune systems to differentiate into IL-17-secreting cells [20]. IL-17-secreting cells vary and express other pathologic cytokines such as TNF-α, IL-22, and IFN-γ. Among these pathologic cytokines, IL-17 and TNF-α are assumed to be direct inductors of arthritis, enthesitis, and gut inflammation [2]. Blocking agents of IL-17 and TNF-α are already used as a therapeutic choice in axial SpA [21]. PIAS3 substantially suppressed IL-17 and TNF-α in the axial joints, peripheral joints, and gut. Therefore, direct inhibition of STAT3 may be a promising therapeutic choice for SpA treatment in the future.

STAT3 is a major transcriptional factor for Th17 differentiation, whereas STAT5 is a major transcriptional factor for Treg differentiation [14]. Activated STAT proteins bind to intracellular genes and influence the epigenetic patterns and specify naïve CD4^+^ T cells to differentiate into certain Th cells [13]. Activated STAT3 stimulates the activation of retinoic acid receptor-related orphan nuclear receptor γt, which is essential for Th17 induction, and suppresses the transcriptional factor Foxp3, which induces Treg differentiation [22]. STAT3 and STAT5 share multiple binding sites on *Il17* loci and compete for binding [22]. The relative ratio of activated STAT3 and STAT5 regulates Th17 cell lineage differentiation [22]. This study revealed that PIAS3 controls the expression of STAT3 and STAT5 molecules and regulates Th17 and Treg cell differentiation.

IL-17-producing cells vary, and many ongoing studies are working towards discovering major cell types that secrete IL-17 and the relative importance of the adaptive/innate immune response in SpA pathogenesis. Myeloperoxidase-positive neutrophils are dominant IL-17-positive cells in the facet joint, whereas c-Kit-positive mast cells comprise the major IL-17-positive cell population in the synovium [23, 24]. A recent study showed that mucosa-associated invariant T (MAIT) cells were elevated in the synovial fluid of patients with AS and presented the IL-17 phenotype [25]. Circulating Th17 cells were elevated in patients with AS and even in patients with early axial SpA [7, 26, 27]. Previous studies have shown that a responder to TNF-α inhibition caused a decrease in circulating Th17 cells and an increase in circulating Treg cells and vice versa in a TNF-α inhibitor non-responder [28]. The transmission of SpA features to SCID mice occurs by transferring CD4^+^ cells from curdlan-induced SpA mice [18]. The major pathologic immune cell in SpA remains uncertain, but Th17 cells are one of the main pathologic cells. STAT3 signaling affects not only Th17 cell differentiation, but also MAIT and NKT cell differentiation [29]. We revealed that PIAS3 has an inhibitory function on Th17 cell polarization and the expression of pathologic cytokines. Although we focused on the Th cell subset in this study, further research studies should determine whether PIAS3 suppresses other IL-17-secreting cells, such as MAIT cells.

The prevention of exaggerated osteoproliferation in axial joints is one therapeutic goal in SpA treatment. Syndesmophyte can occur in the corner of the spine and eventually form bamboo spine. Syndesmophyte formation is followed by several stages: inflammation, erosion, and ankyloses [30]. It is assumed that the main pathologic cytokines differ at each stage, and that Wnt and BMP signaling are the main pathways that act on the final syndesmophyte formation stage [30]. Observational studies have revealed that a TNF-α inhibitor slowed the radiologic damage of the spine in AS, albeit with some limitations [31–33]. First, these results were not from a randomized control trial. Second, enrolled patients were heterogenic in terms of disease duration. Sequential observation of the spinal magnetic resonance imaging study revealed that mature lesions (fatty degeneration) were at a higher risk for developing syndesmophyte than acute inflammatory lesions, and the results supported that SpA patients with mature lesions in the spine were more susceptible to developing syndesmophyte [34]. Third, spinal progression was assessed by the modified Stoke ankylosing spondylitis Spine Score, which is not precise when detecting radiologic progression in AS. The effectiveness of TNF-α inhibition on preventing new bone formation is still under debate, and agents that definitely block spinal progression have not been reported until now [35]. Mesenchymal stem cells from AS patients were superior to those from healthy controls in terms of osteogenic differentiation, and osteogenic differentiation capacity was regulated by BMP-2 and Noggin secretion [36]. Chen et al. reported that AS patients with spinal fusion had higher serum BMP-2, BMP-4, and BMP-7 levels than those without spinal fusion [37]. Recent research studies revealed that BMP may play a pivotal role in the “inflammation-driven syndesmophyte” theory by showing increased expression of BMP genes by TNF-α or IL-1β stimulation [38]. In this study, the expression of BMP and TNF-α was reduced by PIAS3 in spine tissue. Further studies are needed to clarify the potential effects of PIAS3 on exaggerated new bone formation in SpA.

## Conclusions

In conclusion, PIAS3 had beneficial effects in our curdlan-induced SpA mouse model. PIAS3 diminished the histologic scores of peripheral arthritis and gut inflammation, and reduced the expression of pro-inflammatory cytokines in the axial/peripheral joints and the gut. In addition, BMP expression was attenuated by PIAS3 in axial joints, and PIAS3 reciprocally regulated Th17/Treg cell differentiation. These findings suggest that PIAS3 could serve as a therapeutic agent for SpA treatment.

## Abbreviations

Abs: antibodies
APC: allophycocyanin
AS: ankylosing spondylitis
BMP: bone morphogenic protein
CD: cluster of differentiation
FITC: fluorescein isothiocyanate
FOXP3: forkhead box P3
H&E: hematoxylin and eosin
IFN: interferon
IL: interleukin
i.p: intraperitoneal
MAIT: mucosa-associated invariant T
NKT: natural killer T cells
perCP: peridin chlorophyll protein
PIAS3: protein inhibitor of activated STAT3
PE: phycoerythrin
RA: rheumatoid arthritis
SCID: severe combined immunodeficiency
SpA: spondyloarthritis
STAT: signal transducer and activator of transcription
Th: helper T cell
TNF-α: tumor necrosis factor-α
Treg: regulatory T cell
